# Multidimensional impact of fixed-dose subcutaneous trastuzumab-pertuzumab on oncology workflow and patient time burden in a real-world study

**DOI:** 10.1007/s12282-025-01803-6

**Published:** 2025-12-04

**Authors:** Masanori Oshi, Tomo Nakayama, Ichiro Ota, Mahato Sasamoto, Akimitsu Yamada, Kazutaka Narui, Kazuaki Takabe, Itaru Endo

**Affiliations:** 1 Department of Breast Surgery, Graduate School of Medicine, Yokohama City University Hospital, 3-9 Fukuura, Kanazawa ward, Yokohama, 236-0004 Kanagawa Japan; 2 Department of Nursing, Yokohama City University Hospital, Yokohama, Japan; 3 Pharmaceutical Department, Yokohama City University Hospital, Yokohama, Japan; 4 Department of Breast and Thyroid Surgery, Yokohama City University Hospital, Yokohama, Japan; 5 Breast Surgery, Department of Surgical Oncology, Roswell Park Comprehensive Cancer Center, Buffalo, NY USA; 6 Department of Immunology, Roswell Park Comprehensive Cancer Center, Buffalo, NY USA; 7 Department of Surgery, Jacobs School of Medicine and Biomedical Sciences, University at Buffalo, State University of New York, Buffalo, NY 14263 USA

**Keywords:** Economic burden, Healthcare resource utilization, HER2-poistive breast cancer, Phesgo, Subcutaneous administration, Time toxicity

## Abstract

**Background:**

Fixed-dose subcutaneous (SC) trastuzumab and pertuzumab (HP) (Phesgo^®^) offers a clinically non-inferior alternative to intravenous (IV) administration for HER2-positive breast cancer, with potential advantages in treatment efficiency. However, real-world evidence on its impact on healthcare workflow and frontline staff remains limited.

**Methods:**

We conducted a single-center, mixed-methods evaluation comparing SC Phesgo^®^ with conventional IV HP in the perioperative setting. Outcomes included patient treatment time, institutional reimbursement, and the operational burden on nurses and pharmacists, assessed through structured surveys, time-motion observations, and administrative records.

**Results:**

Phesgo^®^ reduced active treatment and clinic visit times by up to 96% and 53%, respectively. Cumulative chemotherapy suite time decreased by 332 h over eight months, indicating a potential for resource reallocation. Nurses and pharmacists reported reduced task complexity and improved workflow satisfaction, particularly during monotherapy. .Although combination regimens introduced procedural complexity, professional motivation remained stable. While SC administration is not eligible for chemotherapy-specific reimbursement add-ons in Japan, efficiency gains may partially offset revenue loss. Patient out-of-pocked costs increased slightly in lower-weight individuals but remained modest overall.

**Conclusions:**

Phasgo^®^ improves both patient time burden and staff workflow efficiency, supporting more sustainable and patient-centered delivery of HER2-targeted therapy. These findings highlight the broader system-level value of fixed-dose SC regimens in high-volume oncology settings under real-world conditions.

**Supplementary Information:**

The online version contains supplementary material available at 10.1007/s12282-025-01803-6.

## Introduction

Breast Cancer remains the most commonly diagnosed malignancy among women worldwide, and its incidence continues to rise. Approximately 20% of cases are classified as human epidermal growth factor receptor 2 (HER2)-positive, a subtype associated with aggressive behavior and poor clinical outcomes [1]. The combination of Trastuzumab and Pertuzumab with chemotherapy has become the standard of care for both early-stage and metastatic HER2-positeive breast cancer [2]. In the perioperative setting, HER2-targeted therapy typically spans up to one year, with longer durations in metastatic cases. However, intravenous (IV) administration of monoclonal antibodies imposes a significant time burden on patients and institutions, first infusions can take over 2.5 h, and subsequent cycles often require 2 h, leading to what is increasingly recognized as “time toxicity” [3].

To address these logistical challenges, a fixed-dose subcutaneous (SC) formulation of trasuzumab and Pertuzumab with hyaluronidase-α, which allows large-volume injection by transiently depolymerizing subcutaneous hyaluronic acid (Phasgo^®^), was approved in 2020, for HER2-positive early and metastatic breast cancer [4]. It eliminates weight-based calculation and markedly shortens administration time, from approximately 150 min for IV HP to around 10 min for SC injection. These features simplify drug preparation, reduce infusion workload, and improve convenience for both patients and healthcare provides. In clinical trials, over 80% of participants preferred SC formulation over intravenous delivery [5]. While previous reports have mainly evaluated efficacy and patient preference, evidence on real-world operational impact, including staff workload and medical economics, remains scarce, particularly in Japan, where the reimbursement framework differs from Western healthcare systems. Despite its clinical non-inferiority, real-world data evaluating its operational impact, particularly on healthcare personnel workflow, reimbursement systems, and outpatient care dynamics, remain limited. This is especially true in Japan, where reimbursement structures differ significantly from western settings.

This study provides the first comprehensive, real-world evaluation of Phesgo^®^ implementation investigating its multidimensional effects on institutional reimbursement, patient time and cost, chemotherapy workflow efficiency, and nursing and pharmacy burden, aiming to inform broader strategies for integrating fixed-dose SC biologics into modern oncology care, and may provide a foundation for evaluating similar innovations in healthcare delivery.

## Materials and methods

### Study design and setting

This was a single-center, retrospective, observational study conducted at a tertiary cancer center in Japan. The evaluation focused on the institutional implementation of fixed-dose subcutaneous trastuzumab and pertuzumab (Phesgo^®^) in patients with HER2-positive breast cancer during the first 8 months following its approval and reimbursement under the Japanese national health insurance system. The study included patients treated in both perioperative and metastatic settings, as intravenous HP use substantially declined after Phesgo^®^ introduction. Because the study objective was to evaluate operational efficiency and economic aspects rather than clinical outcomes, inclusion of both treatment settings was considered appropriate. Patients undergoing other concurrent treatments or examinations during the same visit were excluded.

### Time analysis and patient data

Time-based metrics, including total outpatient stay and active treatment time, were obtained from electronic medical records, nursing logs, and standardized time-motion observation. Active treatment time was defined as the interval from vascular access placement (for IV) or subcutaneous injection preparation (for SC) to catheter or needle removal following drug administration. For Phesgo^®^ patients, all visits during the 8-month study period were included. For the HP cohort, patients receiving neoadjuvant or adjuvant HER2-targeted therapy between 2022 and Phesgo^®^ adoption were selected to match treatment intent and administration protocols. Combination regimens primarily included docetaxel-based chemotherapy in the perioperative setting and standard HER2-directed therapy for metastatic disease.

### Staff survey and workflow assessment

To evaluate the operational impact on frontline personnel, structured questionnaires were administered to nurses and pharmacists involved in HER2-targeted therapy. The surveys included 5-point Likert scale items across multiple domains:

(1) Time burden, (2) Task complexity, (3) Preparation steps, (4) Professional motivation, (5) Perceived patient satisfaction, (6) Workflow stress, (7) Overall impression of regimen.

For nurses, additional items assessed intravenous vs. subcutaneous preparation steps and perceived task load in monotherapy versus combination settings. Pharmacists were also asked to compare aseptic preparation burden, verification workload, and counseling dynamics between HP and Phesgo^®^. Time-motion comparisons were supplemented by observational logging of preparation procedures, step counts, and estimated time per task, recorded during routine clinical operations without intervention.

## Results

### Chemotherapy suite utilization time following Phesgo^®^ implementation

To assess the operational impact of Phesgo^®^ on chemotherapy suite usage, we first calculated the time saved by switching from intravenous HP to fixed-dose subcutaneous Phesgo under actual treatment conditions. Time reductions were estimated using the scheduled chair time allocated for each regimen in our institutional protocols, rather than direct stopwatch-based measurements. Time reductions were estimated separately for monotherapy and combination regimens based on institutional protocols. For monotherapy cases, Phesgo^®^ shortened chemotherapy suite occupancy by approximately 2.0 h during the first cycle and 1.5 h during subsequent cycles. For combination regimens involving docetaxel, the corresponding reductions were 3.0 h for the first cycle and 1.0 h for later cycles. Over an 8-month period following Phesgo^®^ implementation, the drug was administered a total of 157 times at our institution. Using these parameters, the cumulative reduction in chemotherapy suite reservation time was calculated to be 332 h across the 8-month span, corresponding to an average of 41 h per month (Table 1). Assuming that each conventional infusion-based treatment occupies 2.5 h of chair time, this saved capacity could theoretically accommodate up to 132 additional treatment sessions. These estimates represent potential efficiency gains based on scheduled chair times and not incorporate factors such as staffing or room turnover. It should also be noted that this analysis reflects the early phase of Phesgo^®^ implementation at our institution; usage patterns may evolve with increasing familiarity and workflow optimization over time.

**Table 1:** Reduction in appointment slots at the chemotherapy center (infusion appointment times) due to switching from HP to Phesgo^®^

Regimen	IV appointment time (h)	Time saved when scheduling infusions by switching to PHES (h)
HP + docetaxel (first cycle)	5	3
PHES + docetaxel( first cycle)	2	
HP + docetaxel (second and subsequent cycle)	3	1
PHES + docetaxel (Second and subsequent cycle)	2	
HP (first cycle)	3	2
PHES (first cycle)	1	
HP (second and subsequent cycle)	2.5	1.5
PHES (second and subsequent cycle)	1	

Number of cases of PHES regimen implementation (survey period: January 2024 to August)		
Regimen	Number of times	Reduced infusion appointment times (h)
PHES + docetaxel (first cycle)	18	54
PHES + docetaxel (Second and subsequent cycle)	37	37
PHES (first cycle)	11	22
PHES (second and subsequent cycle)	146	219
	Total	332
	PHES single agent only	241

PHES, Phesgo, HP, trastuzumab and pertuzumab

### Patient time burden in hospital-based outpatient care following Phesgo^®^ implementation

**Fig. 1:**
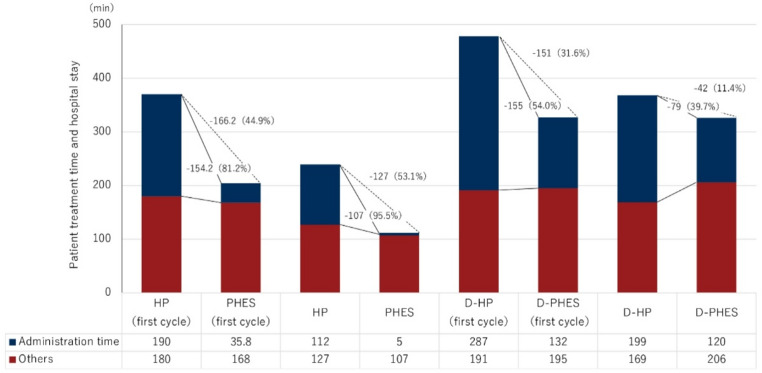
Comparison of outpatient treatment time and total visit duration between Phesgo^®^ and intravenous HP regimens. Total time spent in the outpatient clinic and active treatment duration for patients receiving Phesgo^®^ or intravenous HP, shown separately for monotherapy and combination (with docetaxel) regimens. Clinic time includes all phases from arrival to completion of the visit, encompassing waiting, preparation, administration, and post-treatment observation. Active treatment time refers to the interval between the start and end of drug administration. All durations are expressed in minutes

Although the direct financial implications of Phesgo^®^ versus HP are relevant to healthcare systems and reimbursement models, time burden has increasingly been recognized as a parallel dimension of treatment toxicity, particularly for patients undergoing frequent or prolonged therapy. To evaluate the impact of Phesgo^®^ on patient time burden in the outpatient setting, we conducted a comparative analysis of total clinic visit duration and active treatment time between the subcutaneous Phesgo^®^ regimen and the conventional intravenous HP. Time metrics were assessed separately for monotherapy and docetaxel-combination regimens, and further stratified by initial versus subsequent administrations, recognizing the procedural differences between first-dose protocols and maintenance dosing. In monotherapy settings, Phesgo^®^ was associated with a marked reduction in both treatment-related time components. For the initial administration, active treatment time decreased by 154 min (– 81.2%) and total clinic visit duration was shortened by 166.2 min (– 44.9%) compared to IV HP. For subsequent doses, the reduction remains substantial, with active treatment time decreasing by 108 min (– 95.5%) and visit duration reduced by 127 min (– 53.1%). When administered in combination with docetaxel, Phesgo^®^ also demonstrated a significant time-saving advantage. At the first cycle, active treatment time was reduced by 155 min (– 54.0%), and overall clinic time was shortened by 151 min (– 31.5%). These differences persistent, albeit to a lesser degree, in later cycles: active treatment time decreased by 79 min (– 39.7%) and total visit time by 42 min (– 11.4%). (Fig. 1). These results indicate that Phesgo^®^ consistently reduces time burden in the outpatient setting across both monotherapy and combination contexts, representing a quantifiable operational advantage from the patient perspective.

### Operational impact of Phesgo^®^ on nursing workflow and staff perception

**Fig. 2:**
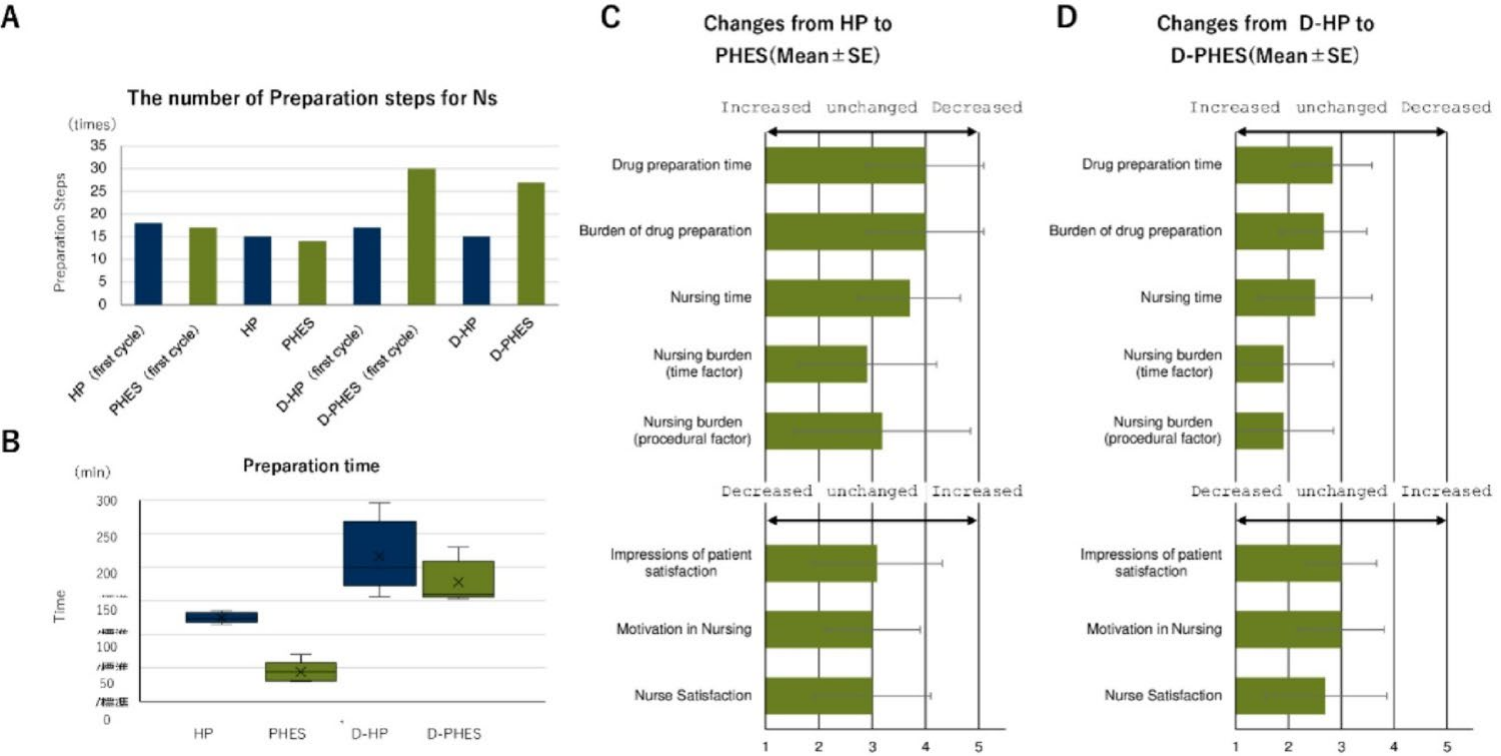
Nursing workflow and staff perception following Phesgo^®^ implementation.** A** Comparison of the number of preparation steps required for intravenous trastuzumab-pertuzumab (HP) therapy versus subcutaneous Phesgo^®^, stratified by monotherapy and combination (with docetaxel) regimens.** B** Total preparation time per administration under each regimen, as recorded in clinical practice.** C** Nurse-reported survey results comparing Phesgo^®^ monotherapy with IV HP monotherapy across eight domains, including time burden, task complexity, patient satisfaction, and overall professional impression. Responses were collected using a 5-point Likert scale.** D** Nurse-reported survey results for combination regimens (Phesgo^®^ plus docetaxel vs. IV HP plus docetaxel), using the same eight-domain, 5-point Likert scale questionnaire

To investigate how subcutaneous HER2-targeted therapy translates into day-to-day clinical operations, we examined the effects of Phesgo^®^ adoption on frontline workflows and staff experience in a high-volume oncology setting. As an initial step, we focused on nursing staff, who are directly responsible for drug administration, patient monitoring, and management of treatment-room operations. This evaluation assessed changes in procedural complexity, preparation time, and professional perception following the transition from conventional intravenous HP therapy to the subcutaneous fixed-dose formulation. Clear differences emerged between monotherapy and combination regimens. When Phesgo^®^ was administered alone, it streamlined nursing procedures, reducing both the number of steps required and the total preparation time (Fig. 2A and B). In contrast, when combined with a taxane, Phesgo^®^ required concurrent subcutaneous injection and intravenous infusion, reintroducing workflow complexity. As expected, this dual route administration increased the number of tasks involved. The overall preparation time remained comparable to that of the conventional HP regimen, despite the increased number of procedural steps. Survey results reflected these workflow patterns. Nurses reported high satisfaction with Phesgo^®^ monotherapy, particularly regarding time efficiency and task simplicity (Fig. 2C). Although combination regimens led to lower scores, especially in domains related to procedural and time burden, nurses’ impressions of patient satisfaction and their own professional motivation remained largely stable (Fig. 2D). Overall, procedural advantage of Phesgo^®^ appeared reduced in multiagent settings, but time efficiency and staff acceptance remained favorable.

### Pharmacy workflow and professional experience following phesgo implementation

Following the nursing evaluation, we extended our analysis to the pharmacy department to examine how Phesgo^®^ adoption influenced preparation workload, verification procedures, and pharmacist perceptions. A structured survey was administered, converting ten domains aligned with those used in the nursing assessment, including technical complexity, time burden, patient interaction, and overall professional satisfaction.

**Fig. 3:**
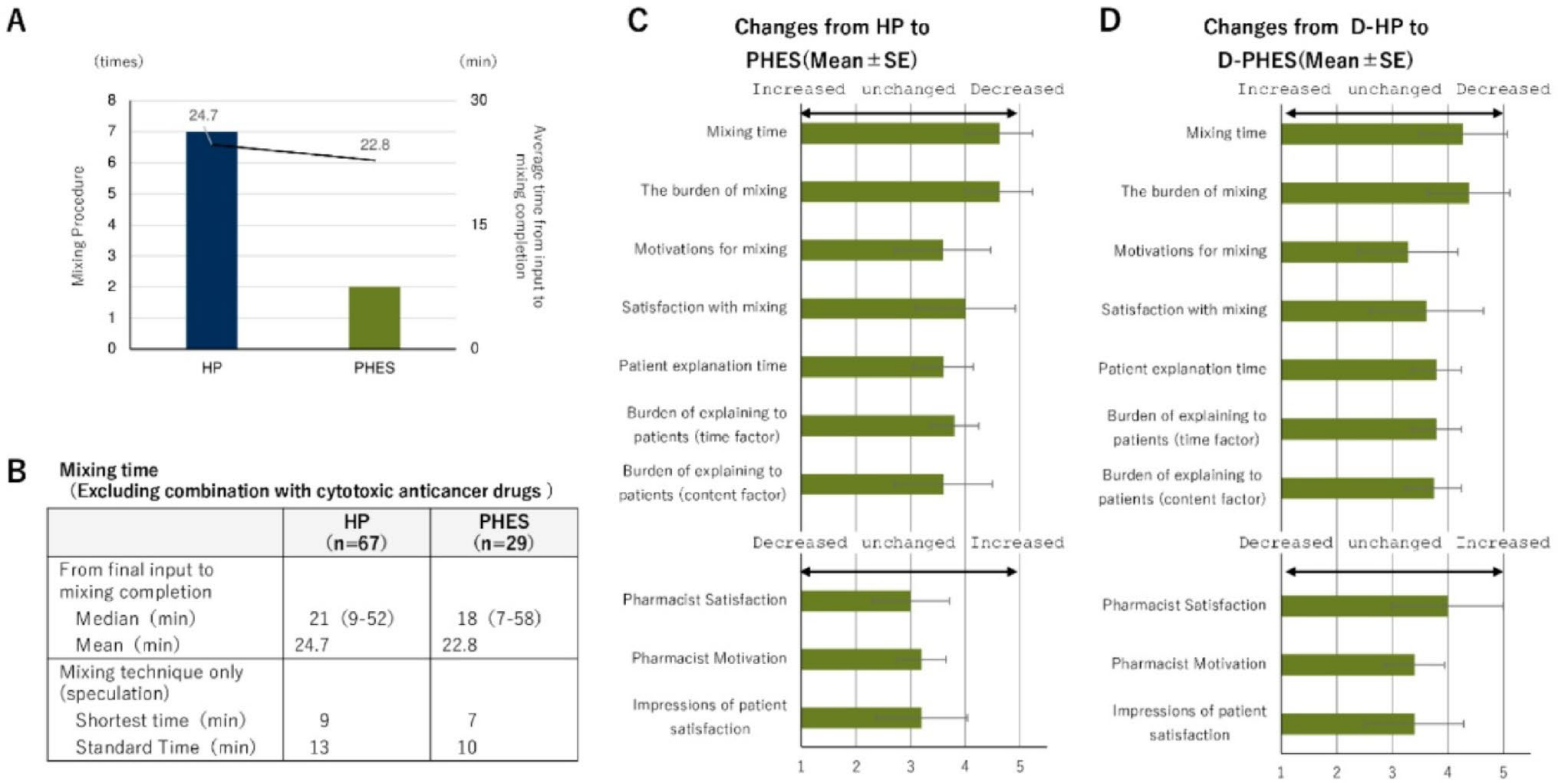
Impact of Phesgo^®^ on pharmacy procedures and professional perception.** A** Comparison of intravenous HP and subcutaneous Phesgo^®^ monotherapy in terms of the number of compounding-related steps (left axis) and average preparation time in minutes, measured from prescription input to mixing completion (right axis).** B** Average total preparation time recorded by pharmacists, measured from prescription input to completion of mixing.** C** Pharmacist-reported survey results comparing Phesgo^®^ monotherapy with HP monotherapy across ten domains, including mixing burden, time efficiency, counseling, and professional motivation. Scores were rated on a 5-point Likert scale (higher values reflect greater favorability toward Phesgo^®^).** D** Pharmacist-reported survey results for combination regimens (Phesgo^®^ plus docetaxel vs. IV HP plus docetaxel), evaluated using the same ten-domain, 5-point Likert scale questionnaire

Pharmacists reported a marked reduction in mixing-related tasks during monotherapy preparations, largely attributable to the elimination of aseptic compounding. When comparing Phesgo^®^ monotherapy with IV HP monotherapy, both the number of preparation steps and the average time from prescription input to mixing completion were reduced (Fig. 3A). Total preparation time, however, remained largely unchanged when comparing monotherapy and combination regimens more broadly (Fig. 3B), suggesting that efficiency gains were concentrated in specific sub-processes without substantially altering the full workflow timeline. Consistent with these findings, survey responses for monotherapy regimens showed high scores for both compounding time and burden (4.53 for each), reflecting favorable impressions of technical efficiency (Fig. 3C). In combination regimens, scores for motivation and satisfaction with compounding were slightly lower (3.29 and 3.62, respectively), likely to reflect the continued need for cytotoxic drug handling. Nevertheless, scores related to patient-centered tasks, such as counseling and perceived patient satisfaction, remained stable or improved modestly. Notably, overall professional satisfaction was higher in the combination setting (4.0) than in monotherapy (3.0), suggesting that pharmacists may derive greater perceived value from their role when engaged in more complex, multi-agent regimens (Fig. 3D). These findings indicate that Phesgo^®^ simplifies technical pharmacy procedures in monotherapy contexts, while preserving or enhancing professional engagement in more complex treatment settings.

### Comparison of patient financial burden between Phesgo^®^ and intravenous HP in perioperativeHER2-positive breast cancer therapy

**Fig. 4:**
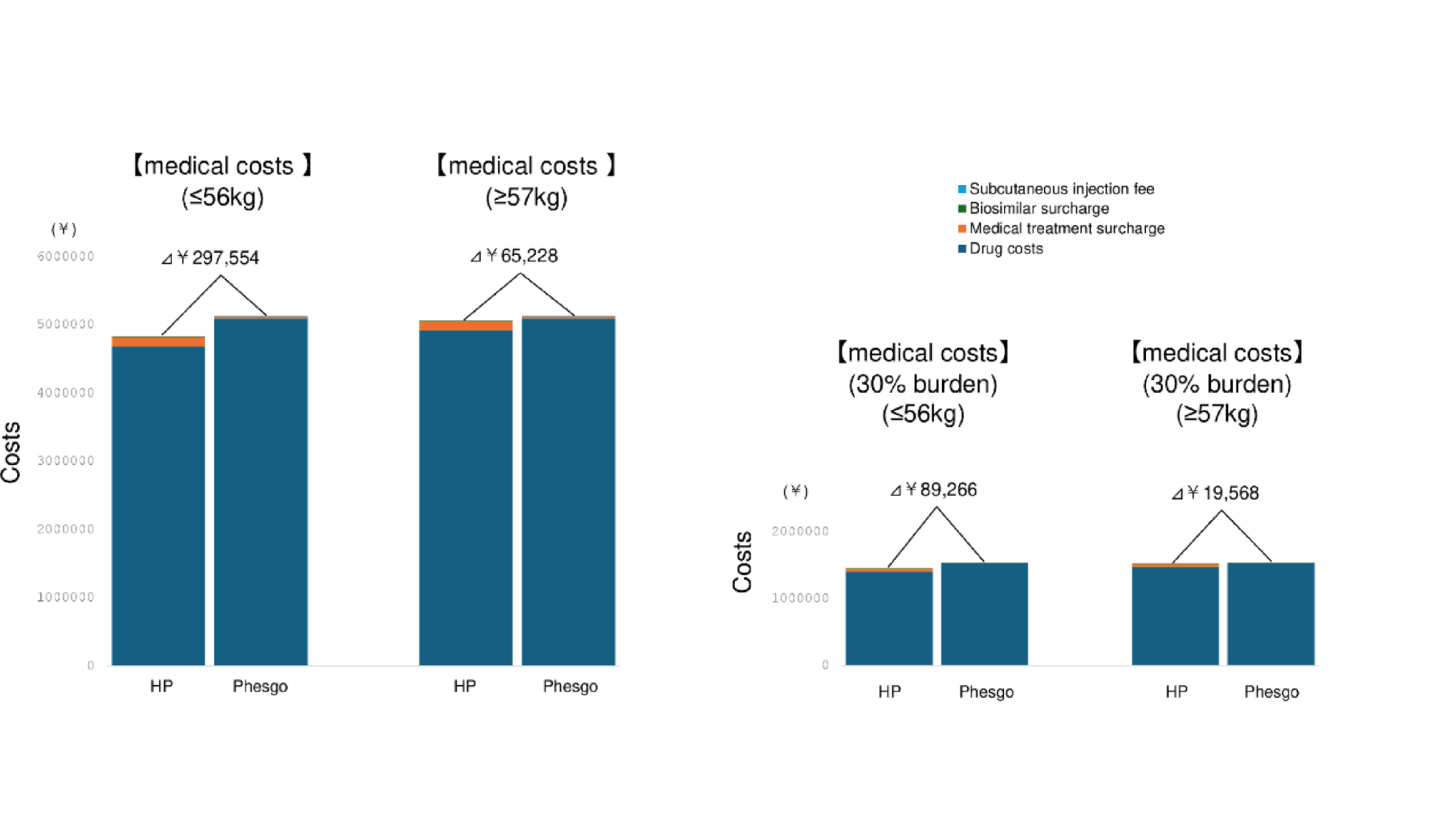
Comparison of cumulative medical costs between intravenous HP and subcutaneous Phesgo^®^ over 18 cycles of perioperative HER2-targeted therapy. The left tall bars illustrate total direct medical costs including drug costs (dark blue), medical treatment surcharges (orange), biosimilar surcharges (green), and subcutaneous injection fees (light blue). The right short bars represent estimated patient out-of -pocket costs assuming a 30% copayment under the Japanese national health insurance system. Cost differences are presented for patients weighing ≤ 56 kg and ≥ 57 kg. NAC and adjuvant phases are stacked to illustrate the total 18-cycle period. Costs are presented in Japanese yen (¥). Approximate U.S. dollar conversions are provided using an exchange rate of ¥150 = US $1 (Bank of Japan rate, October 2025)

As part of a comprehensive evaluation of treatment burden, we assessed the direct financial impact on patients receiving perioperative systemic therapy for HER2-positive breast cancer. This analysis included both neoadjuvant and adjuvant chemotherapy administered alongside trastuzumab and pertuzumab over a total of 18 treatment cycles. Two treatment approaches were compared: conventional intravenous HP and the fixed-dose subcutaneous formulation Phesgo^®^. The cost comparison encompassed four key components: (1) drug costs, (2) outpatient service-related surcharges, (3) biosimilar price differentials, and (4) subcutaneous injection procedure fees. The analysis included drug costs, outpatient chemotherapy service fees, biosimilar surcharges, and subcutaneous injection procedure fees. For patients weighing 56 kg or less, the total medical cost with Phesgo^®^ was \297,554 (approximately US $1,980) higher than that of HP. In patients weighing 57 kg or more, the cost difference was \65,228 (approximately US $435) (Fig. 4). Assuming a standard 30% copayment under the Japanese national health insurance system, the patient out-of-pocket cost for Phesgo^®^ exceeded that of HP by \89,266 (approximately US $595) in the ≦ 56 kg group and \19,568 (approximately US $130) in the ≥ 57 kg group. As drug costs accounted for more than 95% of the total medical expense, we also calculated the per-patient drug cost alone. The drug cost for IV HP was \4,682,642 (approximately US $31,218) for patients weighing ≤ 56 kg and \4,914,968 (approximately US $32,766) for those weighing ≥ 57 kg. In contrast, the drug cost for Phesgo^®^ was \5,095,116 (approximately US $33,967) regardless of body weight. These results demonstrate that while Phesgo^®^ is associated with higher direct medical costs compared to intravenous HP, the relative difference is modest in the context of total treatment expenses and becomes smaller in patients with higher body weight.

### Impact of add-on reimbursement differences on hospital revenue in the perioperative setting for HER2-positive breast cancer

Finally, we evaluated institutional-level reimbursement differences under Japan’s national health insurance system. Although SC injections such as Phesgo^®^ are eligible for a small injection procedure fee, this reimbursement remains substantially lower than the infusion management fee applied to IV regimens. Over the full perioperative treatment course of 18 cycles (including both neoadjuvant and adjuvant phases), the cumulative difference in add-on reimbursement between SC and IV administration corresponds to approximately ¥114,500 (US $760) per patient (Table S1). This difference reflects the current reimbursement structure in Japan, where lower procedural fees for SC administration may reduce institutional incentive for adoption despite clear operational and patient-centered advantages. A detailed breakdown is shown in Fig. 1 and supplementary Table S1.

## Discussion

This study provides the first multidimensional real-world analysis comparing fixed-dose subcutaneous Phesgo^®^ with intravenous HP in the perioperative treatment of HER2-positive breast cancer. Across six domains—including reimbursement structure, treatment time, patient cost and burden, and staff workflow—Phesgo^®^ demonstrated several operational and experiential advantages. Despite lower procedural reimbursement, Phesgo^®^ reduced chemotherapy suite use by over 330 h in 8 months. While modest out-of-pocket cost increases were observed, especially in lighter-weight patients, the overall financial and time impact was minimal, with substantial improvements in efficiency and patient convenience. Staff surveys revealed high satisfaction, particularly among nurses and pharmacists in monotherapy settings, supporting its integration into routine oncology care.

While previous studies have primarily addressed the safety and patient satisfaction for Phesgo^®^ [5, 6], our findings highlight its operational and economic advantages in real-world oncology practice. Although a direct cost comparison may suggest reduced reimbursement due to the absence of injection-related procedural fees, the substantial reduction in chair time, over 330 h within 8 months, demonstrates a distinct efficiency benefit. This improvement in workflow capacity may help accommodate additional patients and optimize treatment scheduling, representing a practical advantage beyond pharmacologic equivalence.

This enhanced efficiency enables institutions to accommodate more chemotherapy sessions and alleviate bottlenecks in outpatient oncology units, an increasingly important issue given the rising number of patients requiring systemic therapy. From an institutional perspective, optimizing treatment throughput may partially offset perceived revenue losses and, in some cases, even generate comparable income through improved capacity utilization. Furthermore, shorter treatment durations improve patient and caregiver convenience, reducing time away from work or daily life. These findings align with global trends toward patient-centered, resource-conscious cancer care, suggesting that the value of subcutaneous formulations such as Phesgo^®^ extends beyond clinical equivalence to strengthening healthcare system resilience under increasing demand.

In terms of patient financial burden, fixed-dose subcutaneous formulations such as Phesgo^®^ may increase out-of-pocket expenses, particularly among lighter-weight patients. In our analysis, patients weighing 56 kg or less incurred an additional cost of approximately ¥89,000 (approximately US $595) compared with intravenous HP. However, this difference represents only a small fraction of the total treatment cost, which also includes chemotherapy, targeted agents, supportive therapies, and diagnostic assessments during the perioperative period. Moreover, Japan’s high-cost medical expense benefit system substantially reduces actual payments for eligible patients, suggesting that the real-world financial impact is minimal and unlikely to influence adherence or treatment choice. Importantly, Phesgo^®^ also provides a marked reduction in chair time, by 40–95% depending on regimen and cycle, helping to offset indirect costs such as lost work hours or caregiver time. These operational efficiencies suggest that, despite modest upfront cost increases, Phesgo^®^ remains a financially feasible and patient-friendly option within Japan’s reimbursement framework. From a national health-economic perspective, the drug cost differential illustrated in Fig. 1 affects not only individual out-of-pocket expenses but also institutional and public healthcare expenditures under Japan’s universal insurance coverage [7, 8]. While the per-patient impact remains modest, cumulative effects could become substantial with broader adoption. Future studies should therefore model the aggregate national expenditure and potential socio-economic benefits of time savings, including downstream effects such as outpatient decentralization, reduced caregiver burden, and productivity gains.

As cancer treatment paradigms grow increasingly complex, healthcare systems face pressure to deliver high-quality care while minimizing operational burden. Fixed-dose subcutaneous formulations like Phesgo^®^ have emerged as a strategy to reduce infusion times and streamline workflows [3]. While prior studies have evaluated subcutaneous delivery in terms of pharmacokinetics and patient preference [9, 10], fewer have examined its real-world operational impact across distinct clinical roles. In this study, we demonstrate that Phesgo^®^ offers tangible workflow advantages over conventional intravenous HP therapy for both nurses and pharmacists, particularly in monotherapy settings. Nurses reported shorter preparation times and reduced procedural complexity, while pharmacists noted substantial decreases in technical workload owing to the elimination of aseptic mixing. These findings align with earlier reports suggesting that subcutaneous administration can improve resource utilization and reduce preparation-related stress [11]. However, our data also reveals a more nuanced picture when Phesgo^®^ is used in combination regimens. Although the procedural burden increased, particularly for nurses who were required to manage both injection and infusion, the overall satisfaction and perception of care quality remained stable. Notably, pharmacists reported higher overall satisfaction with Phesgo^®^ compared to HP even in the combination setting, despite the additional handling demands. This suggests that perceived gains relative to previous practice can outweigh the absolute task volume, highlighting the psychological and experiential dimensions of operational acceptance. What distinguishes this study is its multidimensional evaluation across professional domains, capturing not only technical metrics but also human factors such as perceived workload and professional motivation. To our knowledge, this is the first study to quantify these effects in the Japanese healthcare context, offering insights that are likely generalizable to other high-volume cancer centers with similar operational constraints. Taking together, our results underscore that the impact of subcutaneous fixed-dose therapies such as Phesgo^®^ extends beyond time savings. They influence team-based care dynamics, workflow resilience, and professional satisfaction. Future studies should assess how such regimen affect institutional throughput, staff allocation models, and long-term care delivery outcomes. Tailoring implementation strategies to regimen complexity and role-specific workflows will be essential to fully realize the benefits of subcutaneous oncology treatments.

This study has several limitations. First, it was conducted at a single institution, and clinical and operational context may differ across hospitals and outpatient clinics in Japan. Future investigations should therefore incorporate multi-center data to improve generalizability. Second, patient satisfaction was not assessed, limiting our ability to evaluate patient reported outcomes. While previous clinical trials such as PhranceSCa study have shown high satisfaction with subcutaneous administration, perceptions may vary across care settings and cultures, underscoring the need for domestic patient surveys. Third, safety outcomes were not systematically analyzed; however, no infusion reactions or cardiac dysfunction were observed among patients receiving Phesgo^®^, suggesting favorable tolerability. Fourth, the estimated time savings and treatment capacity gains were based on scheduled regimen times rather than direct time-motion measurements and thus represent idealized operational scenarios. Fifth, these findings may not be directly applicable to other subcutaneous anticancer formulations, which differ in pharmacology and cost structures. Finally, our economic evaluation was limited to direct medical costs under the current Japanese reimbursement system. Broader analyses of cost-effectiveness, long-term resource utilization, and societal economic impact will be important directions for future research.

## Conclusion

This study represents the first comprehensive evaluation comparing intravenous and subcutaneous anti-HER2 therapies for breast cancer across multiple dimensions, including patient time burden, healthcare provider workflow, and institutional financial impact. Given the unique characteristics of Japan’s national insurance system and the variability in clinical operations and staff roles across countries, our findings offer valuable insights specific to the Japanese context. These results underscore the need for similar multidimensional assessments at other institutions to inform broader implementation strategies for subcutaneous anticancer agents in diverse healthcare settings.

## Supplementary Information

Below is the link to the electronic supplementary material.

Supplementary Material 1

## Data Availability

The supporting data, including clinical time logs, cost calculations, and anonymized survey responses from healthcare professionals, are not publicly available due to institutional confidentiality policies. However, de-identified summary data may be made available from the corresponding author upon reasonable request and with permission from the relevant institutional bodies.

## References

[1] Sung H, Ferlay J, Siegel RL, Laversanne M, Soerjomataram I, Jemal A, Bray F. Global cancer statistics 2020: GLOBOCAN estimates of incidence and mortality worldwide for 36 cancers in 185 countries. CA Cancer J Clin. 2021;71:209–49. 33538338 10.3322/caac.21660

[2] Swain SM, Baselga J, Kim SB, Ro J, Semiglazov V, Campone M, Ciruelos E, Ferrero JM, Schneeweiss A, Heeson S, Clark E, Ross G. Benyunes MC and Cortés J. Pertuzumab, trastuzumab, and docetaxel in HER2-positive metastatic breast cancer. N Engl J Med. 2015;372:724–34. 25693012 10.1056/NEJMoa1413513 PMC5584549

[3] Gupta A, Eisenhauer EA, Booth CM. The time toxicity of cancer treatment. J Clin Oncol. 2022;40:1611–5. 35235366 10.1200/JCO.21.02810

[4] Tan AR, Im SA, Mattar A, Colomer R, Stroyakovskii D, Nowecki Z, De Laurentiis M, Pierga JY, Jung KH, Schem C, Hogea A, Badovinac Crnjevic T, Heeson S, Shivhare M, Kirschbrown WP, Restuccia E, Jackisch C. Fixed-dose combination of Pertuzumab and trastuzumab for subcutaneous injection plus chemotherapy in HER2-positive early breast cancer (FeDeriCa): a randomised, open-label, multicentre, non-inferiority, phase 3 study. Lancet Oncol. 2021;22:85–97. 33357420 10.1016/S1470-2045(20)30536-2

[5] O’Shaughnessy J, Sousa S, Cruz J, Fallowfield L, Auvinen P, Pulido C, Cvetanovic A, Wilks S, Ribeiro L, Burotto M, Klingbiel D, Messeri D, Alexandrou A, Trask P, Fredriksson J, Machackova Z, Stamatovic L. Preference for the fixed-dose combination of Pertuzumab and trastuzumab for subcutaneous injection in patients with HER2-positive early breast cancer (PHranceSCa): A randomised, open-label phase II study. Eur J Cancer. 2021;152:223–32. 34147014 10.1016/j.ejca.2021.03.047

[6] Abe T, Sagara A, Suzuki T, Okada D, Takei D, Matsuzaka K, Kobayashi H, Hiraide M, Sano M, Nakayama T. Safety study on switching from intravenous to fixed–dose subcutaneous formulation of Pertuzumab and trastuzumab. Mol Clin Oncol. 2025;22:24. 39885863 10.3892/mco.2025.2819 PMC11775889

[7] McCloskey C, Ortega MT, Nair S, Garcia MJ, Manevy F. A systematic review of time and resource use costs of subcutaneous versus intravenous administration of oncology biologics in a hospital setting. Pharmacoecon Open. 2023;7:3–36. 35996066 10.1007/s41669-022-00361-3 PMC9395845

[8] Ouyang Y, Lee HY, Leong FL, Tey HJ, Shih V, Lim EH, Graves N. Cost-minimization analysis comparing subcutaneous trastuzumab at home with intravenous trastuzumab for HER2-positive breast cancer in Singapore. Ther Adv Med Oncol. 2024;16:17588359241293381. 39529891 10.1177/17588359241293381 PMC11552043

[9] De Cock E, Kritikou P, Sandoval M, Tao S, Wiesner C, Carella AM, Ngoh C, Waterboer T. Time savings with rituximab subcutaneous injection versus rituximab intravenous infusion: A time and motion study in eight countries. PLoS ONE. 2016;11:e0157957. 27362533 10.1371/journal.pone.0157957 PMC4928781

[10] Pivot X, Gligorov J, Müller V, Curigliano G, Knoop A, Verma S, Jenkins V, Scotto N, Osborne S, Fallowfield L. Patients’ preferences for subcutaneous trastuzumab versus conventional intravenous infusion for the adjuvant treatment of HER2-positive early breast cancer: final analysis of 488 patients in the international, randomized, two-cohort prefher study. Ann Oncol. 2014;25:1979–87. 25070545 10.1093/annonc/mdu364

[11] Rummel M, Kim TM, Aversa F, Brugger W, Capochiani E, Plenteda C, Re F, Trask P, Osborne S, Smith R, Grigg A. Preference for subcutaneous or intravenous administration of rituximab among patients with untreated CD20 + diffuse large B-cell lymphoma or follicular lymphoma: results from a prospective, randomized, open-label, crossover study (PrefMab). Ann Oncol. 2017;28:836–42. 28031173 10.1093/annonc/mdw685

