# Supplementary material for: Multidimensional impact of fixed-dose subcutaneous trastuzumab-pertuzumab on oncology workflow and patient time burden in a real-world study
Source: Breast Cancer. 2025 Dec 4;33(1):288–96. doi: 10.1007/s12282-025-01803-6 (PMC12789160; doi:10.1007/s12282-025-01803-6)
Supplement: Supplementary file 1 — Supplementary Material 1 [file 12282_2025_1803_MOESM1_ESM.docx]

**Supplemental table**

**Table S1. Comparison of Perioperative Treatment Reimbursement for HER2-positive Breast Cancer in Japan: HP regimen vs. Phesgo**^®^**.**

| **Additional payment**  PHES：Calculated subcutaneous injection management fee based on medical fee 2024.6 | | |
| --- | --- | --- |
| Injection management fee | Medical fee points |  |
| Subcutaneous injection management fee | 25 |  |
| Intravenous injection management fee  (≦500mL) | 53 |  |
|  |  |  |
| HP：Calculated intravenous injection management fee + treatment fee 1 of outpatient with tumor chemotherapy | | |
| Treatment fee 1 of outpatient with tumor chemotherapy | | Medical fee points |
| Until May 2024 | Every time | 700 |
| After the revision of medical fees  (after 1 June 2024) | 1st to 3rd times in the same month | 800 |
|  | 4th time or more | 450 |
|  |  |  |
| Adjuvant therapy |  | Medical fee points |
| PHES： 25 points×18 times |  | **450** |
| HP+Docetaxel→　　　PHES：800×4+25×14 | | **3,550** |
| HP：800×18+150×4 | | **15,000** |

PHES, Phesgo; HP, trastuzumab and pertuzumab.
